# Semi-field experiments highlight the importance of maize and rice pollen on oviposition site choice and larval development in *Anopheles arabiensis*

**DOI:** 10.1186/s13071-025-07062-w

**Published:** 2025-10-28

**Authors:** Hudson Onen, Emmanuel W. Kaindoa, Perpetra Akite, Jonathan K. Kayondo, Martha A. Kaddumukasa, Anne M. Akol, Frederic Tripet

**Affiliations:** 1 Department of Zoology, Entomology and Fisheries Sciences, College of Natural Sciences, School of Biosciences, Makerere University, P.O Box 7062, Kampala, Uganda; 2 Department of Entomology, Uganda Virus Research Institute, Plot 51/59 Nakiwogo Road, P.O. Box 49, Entebbe, Uganda; 3 Department of Environmental Health and Ecological Science, Ifakara Health Institute, P. O. Box 53, Ifakara, Tanzania; 4 Faculty of Science, Biological Sciences, Kyambogo University, P.O. BOX 1, Kampala, Uganda; 5 Swiss Tropical and Public Health Institute, Kreuzstrasse 2, 4123 Allschwil, Switzerland; 6 University of Basel, Petersplatz 1, 4001 Basel, Switzerland

**Keywords:** *Anopheles gambiae* s.l., Aquatic habitats, Pollen, Semi-field, Oviposition

## Abstract

**Background:**

Members of the *Anopheles gambiae* complex, such as *Anopheles gambiae* sensu stricto (*An. gambiae* s.s.), *Anopheles coluzzii* and *Anopheles arabiensis*, are among the key malaria vectors in sub-Saharan Africa. These species are often abundant in areas of intense rice and maize farming with temporary water pools reflecting the dependence of their larvae on the pollen shed in such pools as food. In this study we explored the oviposition preference of wild-caught gravid *An. arabiensis* in response to maize and rice pollen in artificial aquatic habitats in a semi-field system.

**Methods:**

Twelve experimental breeding habitats were established in each of the two large compartments of a semi-field system. Rice or maize pollen was added into eight randomly selected habitats in eachcompartment; the remaining four habitats of each compartment were used as control habitats without pollens. In the first experiment, 40 gravid *An. arabiensis* were released in each compartment and left overnight to choose egg-laying habitats, following which the eggs were sampled and counted. The second experiment differed from the first experiment only in that the counted eggs were returned to the respective habitat where the development of the resultant larvae was monitored and recorded until pupation.

**Results:**

Pollen types strongly affected the oviposition behaviour of gravid *An. arabiensis*. Females preferred to lay eggs in habitats with rice pollen on the water surface over those with maize pollen, and in habitats with maize pollen on the water surface over pollen-less controls. The development of larvae was significantly affected by the type of pollen in the habitats. The highest total number of *An. arabiensis* offspring were produced in habitats with rice pollen compared to those with maize pollen and no pollen. However, larval development success was comparatively lower in habitats containing rice pollen than those with maize pollen and no pollen, suggesting that the habitats with rice pollen were overcrowded.

**Conclusion:**

This study demonstrates that pollen types on the surface of aquatic habitats influence the oviposition site selection behaviour of gravid *An. arabiensis* and has carry-over effect on the developmental success of their offspring.

**Graphical abstract:**

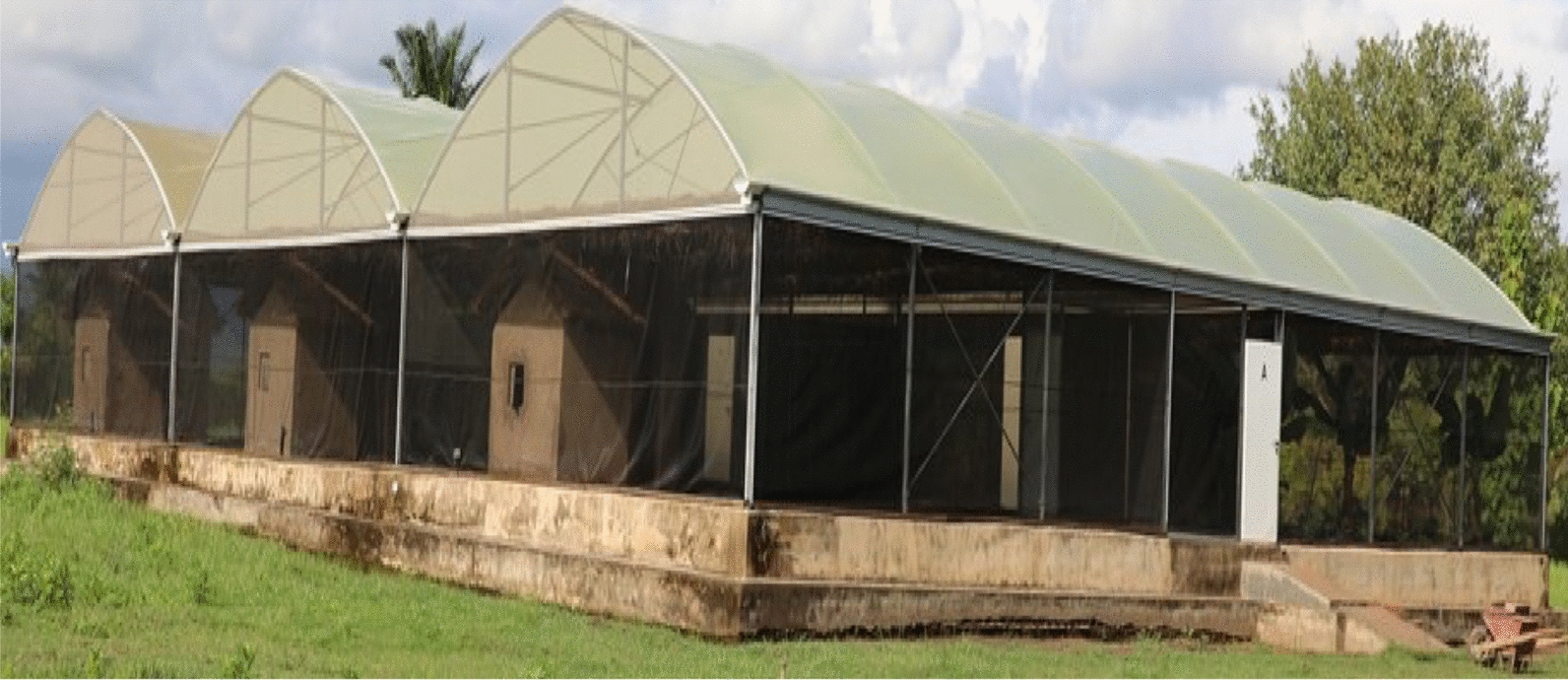

## Background

Species of the Anopheles gambiae sensu lato (*An. gambiae *s.l.) complex, such as *An. gambiae* sensu stricto (*An. gambiae* s.s.), *Anopheles coluzzii* and *Anopheles arabiensis*, are among the main malaria vectors in sub-Saharan Africa [1, 2]. The abundance and distribution of these species are closely linked to seasonal rainfall patterns and the availability of aquatic habitats such as temporary water pools [3, 4]. Adult female mosquitoes tend to aggregate near oviposition sites, increasing the risk of malaria transmission in such localities [5, 6]. Thus, oviposition behaviour is one of the factors explaining the heterogeneous malaria vector distribution across landscapes [5].

The choice of a particular aquatic habitat for oviposition by female mosquitoes is driven by several factors, including the availability of larval food for the offspring [7, 8]. Unlike the larvae of other mosquito genera, such as *Culex* and *Aedes* mosquitoes, that aggregate below the surface of water while feeding, *An*. *gambiae* s.l. larvae are interfacial feeders that preferentially feed on organic particles suspended either in the water column or on its surface [9]. Although most mosquito larvae can feed on particles of any size, particles of certain sizes are ingested more readily than others [10]. Pollen grains are easily ingested by anopheline larvae, which are well-adapted to surface feeding. Anopheline larvae have specialized spiracles that allow them to breathe while floating on the water surface, and they possess the unique ability to rotate their heads 180 degrees, enhancing their ability to skim and filter food such as pollen from the water surface [11–13].

The respective abundances of *An. arabiensis,* which is distributed throughout sub-Saharan Africa, and of *An. coluzzii*, which is restricted to West Africa, often correlate with the presence of transient puddles created as a result of human activities, including rice cultivation [14–17]. The use of habitats associated with rice fields by ovipositing female *An. arabiensis* and *An. coluzzii* is thought to have led to an affinity for grasses from the Poacae family [18, 19]. This affinity has been attributed to the shedding of pollen from these grasses onto the water surface of the aquatic habitats, potentially constituting an important larval food for their offspring. The amount of nutrients available in an aquatic larval habitat is critical for larval survival and development, which determines adult body size and metabolic reserves upon emergence and directly impacts mosquito vectorial capacity [20].

Not only rice cultivation but also maize cultivation is common in most rural villages of Africa, and maize pollen is a source of nutrients for *An. gambiae* s.l. larvae that enhance their development [21]. In typical East African villages, maize and rice plants are grown near human homesteads throughout the year. The cultivation of these plants is conducted on the uplands during wet seasons and in swampy areas during the dry seasons. Mature maize and rice plants produce a copious amount of nutritious wind-borne pollen. Their pollen grains are relatively large (34–90 μm) and thin-walled, which limits their dispersal range and causes them to become trapped in the surface film of water, on which they float. Both rice and maize plants bloom over a sufficiently long period to support the development of at least one generation of anopheline mosquitoes [11, 22]. Thus, the mode of maize and rice cultivation likely provides a constant pollen supply as mosquito larval food, thereby sustaining high population of malaria vectors and malaria prevalence.

It has been suggested that the adaptation of *An. gambiae* s.l. larvae to feeding on rice and maize pollen is a recent adaptation [21, 23], based on knowledge that rice domestication started 10,000 years ago in the Niger River Basin and its cultivation quickly spread across West and Central Africa [24]. Domestic cultivation focused on the African rice (*Oryza glaberrima*) until the introduction of Asian rice (*Oryza sativa*) in the fifteenth to sixteenth centuries [24]. Maize was introduced to tropical Africa by the Portuguese in the sixteenth century and was soon widely grown along the coast from the Gambia River to Sao Tomé, situated at the mouth of the Congo River, and possibly in Ethiopia [25].

The risks associated with ephemeral aquatic habitats, long droughts and absence of larval food in the aquatic habitats make cautious oviposition site selection by a female mosquito a critical step [26] and suggests that gravid females should have the ability to select oviposition sites with sufficient larval food that enhance the development of their offspring. Laboratory and cage experiments have demonstrated that gravid *An. arabiensis* are attracted and oviposit in response to odour emanating from maize and rice pollen [27, 28]. Indeed, (1R)-(+)-α-pinene and nonanal compounds are both components of rice and maize pollen that are attractive to gravid mosquitoes [19]. These allomones have been tested as attractants in mosquito gravid female traps, with the results indicating that they can further be explored for use as vector control tools [29]. Currently, there are few or no direct comparisons of the attraction of female *An. arabiensis* to habitats inoculated with maize and rice pollen as well as pollen-less aquatic habitats at a spatial scale relevant to natural oviposition site searches, such as large semi-field enclosures. In addition, no studies have examined the fitness consequences of such female oviposition site choice by measuring its carry-over effects on successful larval development. The lack of such experiments makes it difficult to effectively evaluate the adaptive value of female oviposition choice.

In this study, we hypothesized that gravid *An. arabiensis* adaptively prefer aquatic oviposition sites with rice and maize pollen over those with no pollen because the former have the potential to provide food for their larval offspring. To test this hypothesis, oviposition site preference was experimentally compared by releasing wild-caught gravid *An. arabiensis* in a large semi-field enclosure with randomized artificial habitats containing rice and maize pollen as well as control habitats without pollen. These data will contribute to our understanding of the importance of larval food availability in aquatic habitats as a factor used by gravid female *An. arabiensis* to assess the suitability of the aquatic habitats for their offspring. Such knowledge is important for novel vector control applications that may make use of attractants derived from these pollens for new vector control tools.

## Methods

### Study area

The study was conducted between March and October 2022 in semi-field system of the Ifakara Health Institute (IHI) located in Kining’ina village (8°07′18″S, 36°39′06″E), a small farming town situated approximately 5 km north of Ifakara town [31]. Details of the semi-field system (SFS) are described in details in previous studies [31–33].

### Artificial aquatic habitats

Twelve black-coloured basins (diameter: 30 cm, capacity: 20 l) were firmly planted in each of the two compartments  of the SFS following a randomized Latin square design of 3 rows × 4 columns, following which 10 g of the adjacent sieved soil and available well water were added to each basin; these basins were used as aquatic habitats [32]. The set-up was left for 48 h to acclimatize to the SFS conditions.

### Pollen collection

Rice and maize pollens were collected between 7:00 h and 10:00 h from farms located in Mahutanga (8°08′29″S, 36°39′21″E), Michenga (8°07′07″S, 36°39′11″E) and Kining'na (8°07′18″S, 36°39′06″E). These villages experience high malaria prevalence owing to the abundance of *An. arabiensis* and *An. funestus* [34]. During the collection period, a tassel of each plant type was inserted into a transparent Ziplock polythene bag and gently shaken to dislodge the pollen. The collected pollen was immediately transported to the IHI laboratory and stored for 3 days at − 20 °C [35].

### Collection of blood-fed female mosquitoes

Wild blood-fed female mosquitoes were collected using mouth aspirators from accessible huts in Tulizamoyo (− 8.35447°S, 36.70546°E) and Lupiro (− 8.38°S, 36.67°E) villages, both situated in Ulanga district (8.9802°S, 36.7820°E) as previously described [32]. *Anopheles arabiensis* is the sole species of the *An. gambiae* complex found in these locations. *Anopheles gambiae* s.s. mosquitoes have progressively disappeared from these locations over the past two decades [36, 37], and their absence was further confirmed during this study by separately characterizing over 3000 larvae produced from field-collected female eggs batches by DNAzol® PCR diagnostics [38], with the results confirming only *An. arabiensis* was present in these locations. Subsequently, *An. arabiensis* females were simply identified using morphological characters. The collected females were transferred into the field collection cages (15 × 15 × 15 cm), provided with a 10% sugar solution, transported to IHI semi-field insectary and later kept in those cages for 3 additional days to become gravid and ready for oviposition.

### Experiment 1: egg-laying choice

From the stored pollen, samples (0.5 g) of each pollen type were weighed out and separately wrapped in aluminium foil. These were then transported to the semi-field site on the same day, where the water in each habitat was randomly sprinkled with maize or rice pollen, with the exception of control habitats without pollen.

Two sets of 40 physically gravid mosquitoes were aspirated from the field collection cages using a mouth aspirator. Each set was then transferred into a paper cup through a hole punched on the non-insecticide-treated mosquito net used as a lid, and the hole then plugged with cotton wool. The mosquitoes were allowed to settle for 1 h while provided with 10% sugar solution. Each mosquito set was released into eachof the two randomly selected compartments of the SFS. Twenty-four hours later, mosquito eggs were searched on the water surface of the habitats using a magnifying glass and a head torch. All eggs were collected by sliding filter papers on the water-wall interface of the habitats. The eggs collected from each habitat category were transferred into a respective petri dish inoculated with moist filter papers. The Petri dishes were subsequently labelled with a unique habitat code indicating where the eggs were collected and transported to the IHI’s Vector sphere laboratory for counting. To reduce bias during data entry, a trained technician re-assigned new codes to the Petri dishes containing eggs (1–24) using labels that covered the codes assigned during egg collection. The eggs were then counted under the dissecting microscope at ×40 magnification and later discarded. The presence and number of eggs laid in each habitat category were used as indicators of habitat preference.

### Experiment 2: larval developmental success

A similar procedure as described above was followed, except that the counted eggs were returned to each habitat (bucket) where they were collected and the resultant larvae were left to grow with the pollen without any additional provision of larval food. During the period of monitoring larval development, larvae and/or pupae were sampled from each habitat using a 3-ml Pasteur pipette and counted on days 1 (24 h), 2 (48 h), 3 (72), 4 (96 h), 5 (120 h) and 6 (144 h). On each day, all of the counted larvae were returned to their respective habitats, while pupae were counted and killed using 100% ethanol and discarded. The experiments were repeated 3 times, with habitats emptied and cleaned between each experiment.

### Data analyses

Data were analysed using JMP version 14 software (SAS Institute, Inc., Cary, NC, USA) after checking for deviations from normality and heterogeneity. The analyses were conducted using parametric and non-parametric methods as appropriate. Data from all replicates were used for the analysis, and replicate effects were tested but were only reported when significant or when the interaction with any other main effect was significant. Interactions between independent variables were tested using stepwise models, and only significant variables were retained in the final models. Stepwise generalized linear regression using Poisson distribution with log link function and checked-over dispersion was used to determine factors influencing the numbers of eggs laid by gravid mosquitoes across pollen types in the habitats. Post-hoc pairwise comparisons were used to further compare differences in the number of eggs laid by gravid *An. arabiensis* across pollen types. Stepwise nominal logistic regression was used to determine the effects of pollen types on larval developmental success rate across habitats, and likelihood odds ratios was used for post-hoc pairwise group comparisons. Due to the possibility of counting the same individuals between successive days, which would lead to inflated numbers, all eggs laid in habitats were counted on day 1 and considered for analysis. The total number of offspring successfully produced per habitat was calculated as the sum of all pupae produced (collected on days 5 and 6) and all fourth-larval instar remaining in habitats on day 6 (final day of experiment).

## Results

### Experiment 1: Egg-laying choice of female *An. arabiensis*

#### Effect of pollen type on habitats used

**Fig. 1:**
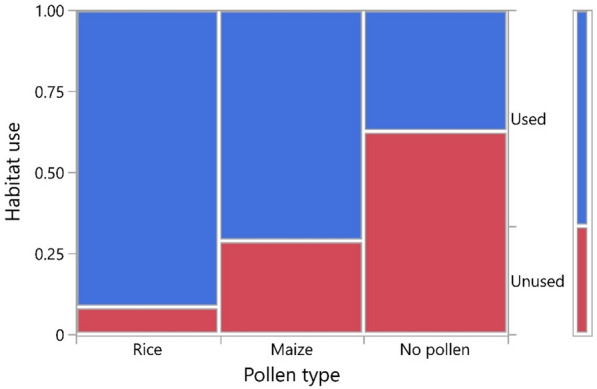
Proportion of habitats used by gravid *Anopheles arabiensis* females across experimental treatment groups

Overall, 66.7% of habitats were used for oviposition by gravid *An. arabiensis* in the first experiment. Nominal logistic regression showed that habitat use was significantly affected by the type of pollen present in the aquatic habitats (L-R chi-square  17.45,* n* = 72, *p* = 0.002), but not by the semi-field compartment used (L-R chi-square 1.298,* n* = 72, *p* = 0.255). The proportion of aquatic habitat used did not statistically differ between those with rice and maize pollen (odds ratio [OR] 4.629, *p* = 0.057) (Fig. 1). Control habitats without pollen were used significantly less compared to those inoculated with either pollen type (OR > 4.195, *p* < 0.0180 in both cases).

#### Effect of pollen type on number of eggs laid

Overall, the highest number of eggs laid occurred in habitats with rice pollen (*n* = 2908 eggs), followed by maize (*n* = 1673) and, lastly, by habitats with no pollen (*n* = 546) (Table 1). The mean number of eggs collected per habitat, irrespective of pollen type and compartment, after 1 night of female oviposition was 71.2 (95% confidence interval [CI] 57.1–85.2) (Table 1).

**Table 1:** Habitat use, mean and total number of eggs laid by gravid *Anopheles arabiensis* females across pollen types, replicates and compartments

Treat/replicates	Compartment 1				Compartment 2				Both compartments			
	*N*	% Habitat used (95% CI)	Mean eggs,* n* (95% CI)	Total eggs,* n*	*N*	% Habitat used (95% CI)	Mean eggs,* n* (95% CI)	Total eggs,* n*	*N*	% Habitat used (95% CI)	Mean eggs,* n* (95% CI)	Total eggs,* n*
*Replicate 1*												
No pollen	4	50 (15.0–85.1)	30 (− 37.5 to 97.5)	120	4	50 (15.0–85.1)	25.8 (− 22.6 to 74.1)	103	8	50 (21.5–78.5)	27.9 (− 0.7 to 56.5)	223
Maize pollen	4	75 (30.1–95.4)	49.8 (− 3.7 to 103.2)	199	4	75 (30.1–95.4)	93.8 (− 7.1 to 194.6)	375	8	75 (41.0–92.9)	71.8 (27.8–115.7)	574
Rice pollen	4	100 (51.0–100	113.8 (84.7–142.8)	455	4	100 (51.0–100	175.8 (125.5–226.0)	703	8	100 (67.6–100)	144.8 (110.6–178.9)	1158
All treatments	12	75 (46.8–91.1)	64.5 (34.1–94.9)	774	12	75 (46.8–91.1)	98.4 (50.4–146.5)	1181	24	75 (55.1–88.0)	81.5 (54.3–108.6)	1955
*Replicate 2*												
No pollen	4	25.0 (4.6–69.9)	12.3 (− 26.7 to 51.2)	49	4	50 (15.0–85.1)	43.3 (− 47.9 to134.5)	173	8	37.5 (13.7–69.4)	27.8 (− 9.1 to 64.6)	222
Maize pollen	4	50 (15.0–85.1)	50.8 (− 42.6 to 144.1)	203	4	75 (30.1–95.4)	70 (− 5.9 to 145.9)	280	8	62.5 (30.6–86.3)	60.4 (18.1–102.6)	483
Rice pollen	4	75 (30.1–95.4)	89 (− 9.9 to 187.9)	356	4	100 (51.0–100	128.8 (92.2–165.3)	515	8	87.5 (52.9–97.8)	108.9 (68.5–149.3)	871
All treatments	12	50.0 (25.4–74.6)	50.7 (14.6–86.8)	608	12	75 (46.8–91.1)	80.7 (45.6–115.8)	968	24	62.5 (42.7–78.8)	65.7 (41.6–89.7)	1576
*Replicate 3*												
No pollen	4	25.0 (4.6–69.9)	10 (− 21.8 to 41.8)	40	4	25.0 (4.6–69.9)	15.3 (− 33.3 to 63.8)	61	8	25 (7.1–59.1)	12.6 (− 7.5 to 32.7)	101
Maize pollen	4	75 (30.1–95.4)	71.8 (− 14.4 to 157.9)	287	4	75 (30.1–95.4)	82.3 (− 8.6 to 173.1)	329	8	75 (41.0–92.9)	77 (33.7–120.3)	616
Rice pollen	4	75 (30.1–95.4)	91.5 (− 11.7 to 194.7)	366	4	100 (51.0–100	128.3 (97.8–158.7)	513	8	87.5 (52.9–97.8)	109.9 (69.4–150.4)	879
All treatments	12	58.3 (32.1–80.7)	57.8 (20.9–94.6)	693	12	66.7 (39.1–86.2)	75.3 (37.2–113.3)	903	24	62.5 (42.7–78.8)	66.5 (41.8–91.2)	1596
*All replicates*	36	61.1 (44.9–75.2)	57.6 (39.7–75.6)	2075	36	72.2 (56.0–84.2)	84.8 (63.4–106.1)	3052	72	66.7 (55.2–76.5)	71.2 (57.1–85.2)	5127

*CI *Confidence interval

**Fig. 2:**
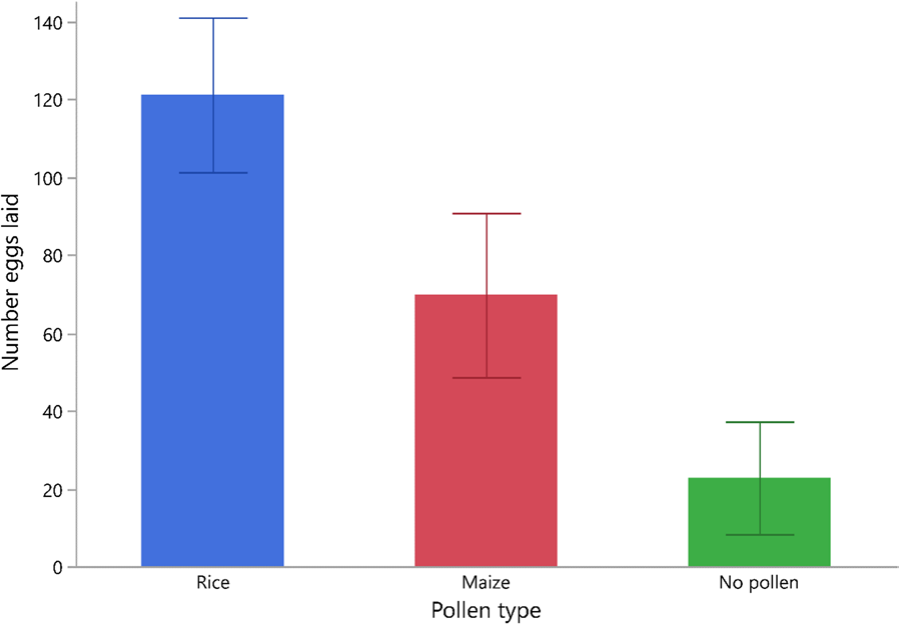
Mean number of eggs laid by gravid *Anopheles arabiensis* females across pollen types

A generalized linear model (Poisson distribution with over-dispersion) showed that there was a significant effect of pollen type (L-R chi-square 53.1, *n* = 72, *p* < 0.001) and compartment on the mean number of eggs laid across habitats (L-R chi-square 5.6, *n* = 72, *p* = 0.018). Post-hoc pairwise comparisons showed that significantly more eggs were laid in habitats with rice pollen than in those with maize pollen, and that the fewest eggs were laid in the control group (model contrast: *χ*^2^ > 17.96, *p* < 0.001 in both cases) (Fig. 2).

### Experiment 2: egg laying choice and larval developmental success

#### Effect of pollen type on habitats used

**Fig. 3:**
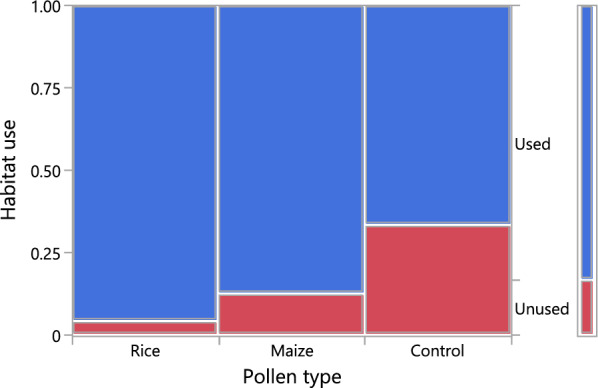
Proportion of habitats used by gravid *Anopheles arabiensis* females across treatment groups

Overall, 83.3% of habitats were used for oviposition in this experiment. A nominal logistic regression showed that habitat use by gravid female *An. arabiensis* was significantly affected by pollen type (L-R chi-square 7.978, *n* = 72, *p* = 0.019) but not by compartment (L-R chi-square 0.450, *n* = 72, *p* = 0.502). Control habitats without pollen were significantly less used for oviposition than the pollen-inoculated habitats (OR < 11.671, *p* = 0.027 in both cases). However, there was no significant difference in habitat preference between those inoculated with rice pollen and those inoculated with maize pollen (*p* = 0.285) (Fig. 3).

#### Effect of pollen type on number of eggs laid

Overall, the highest total number of eggs were laid in habitats containing rice pollen (*n* = 4604), followed by maize pollen (*n* = 2356) and those without pollen (*n* = 1417) (Table 2). The mean number of eggs laid per habitat, irrespective of pollen type and compartment, after 1 night of female oviposition was 116.3 (95% CI 97.9–134.7) (Table 2).

**Table 2:** Habitat use and mean and total number of eggs laid by gravid *Anopheles arabiensis* females across pollen types, replicates and compartments

Treatment/replicate	Compartment 1				Compartment 2				Both compartments			
	*N*	% Habitat used (95% CI)	Mean eggs,* n* (95% CI)	Total eggs,* n*	*N*	% Habitat used (95% CI)	Mean eggs,* n* (95% CI)	Total eggs,* n*	*N*	% Habitat used (95% CI)	Mean eggs,* n* (95% CI)	Total eggs,* n*
*Replicate 1*												
No pollen	4	50 (15.0–85.1)	36.3 (− 33.7 to 106.2)	145	4	75 (30.1–95.4)	57.5 (− 15.6 to 130.6)	230	8	62.5 (30.6–86.3)	46.9 (10.8–82.9)	375
Maize pollen	4	100 (51.0–100)	110.5 (75.2–145.8)	442	4	100 (51.0–100)	107 (95.2–118.8)	428	8	100 (67.6–100)	108.8 (95.9–121.6)	870
Rice pollen	4	100 (51.0–100)	180 (141.2–218.8)	720	4	100 (51.0–100)	223.3 (126.8–319.7)	893	8	100 (67.6–100)	201.6 (160.9–242.3)	1613
All treatments	12	83.3 (55.2–95.3)	108.9 (65.9–151.9)	1307	12	91.7 (64.6–98.5)	129.3 (76.6–181.9)	1551	24	87.5 (69.1–95.7)	119.1 (87.5–150.6)	2858
*Replicate 2*												
No pollen	4	75 (30.1–95.4)	73.5 (− 5.3 to 152.3)	294	4	75 (30.1–95.4)	79 (− 6.1 to 164.1)	316	8	75 (40.9–92.9)	76.3 (36.3–116.2)	610
Maize pollen	4	100 (51.0–100)	119 (78.5–159.5)	476	4	50 (15.0–85.1)	60.5 (− 51.6 to 172.6)	242	8	75 (40.9–92.9)	89.75 (41.1–138.4)	718
Rice pollen	4	100 (51.0–100)	146 (82.1–209.9)	584	4	100 (51.0–100)	212.8 (80.9–344.6)	851	8	100 (67.6–100)	179.4 (120.8–237.9)	1435
All treatments	12	91.7 (64.6–98.5)	112.8 (82.6–143.1)	1354	12	75.0 (46.8–91.1)	117.4 (57.1–177.8)	1409	24	83.3 (64.1–93.3)	115.1 (84.1–146.2)	2763
*Replicate 3*												
No pollen	4	50 (15.0–85.1)	46.3 (− 38.9 to 131.4)	185	4	75 (30.1–95.4)	61.8 (− 8.0 to 131.5)	247	8	62.5 (30.6–86.3)	54 (15.5–92.5)	432
Maize pollen	4	75 (30.1–95.4)	81.5 (− 5.4 to 168.4)	326	4	100 (51.0–100)	110.5 (100.3–120.7)	442	8	87.5 (52.9–97.8)	96 (63.2–128.8)	768
Rice pollen	4	75 (30.1–95.4)	159.3 (− 51.9 to 370.4)	637	4	100 (51.0–100)	229.8 (134.1–325.4)	919	8	87.5 (52.9–97.8)	194.5 (108.8–280.2)	1556
All treatments	12	66.7 (39.1–86.2)	95.7 (36.1–155.3)	1148	12	91.7 (64.6–98.5)	134 (81.0–186.9)	1608	24	79.2 (59.5–90.8)	114.8 (77.2–152.4)	2756
*All replicates*	36	80.6 (65.1–90.2)	105.8 (81.9–129.7)	3809	36	86.1 (71.3–93.9)	126.9 (98.1–155.7)	4568	72	83.3 (73.1–90.2)	116.3 (97.9–134.7)	8377

*CI *Confidence interval

**Fig. 4:**
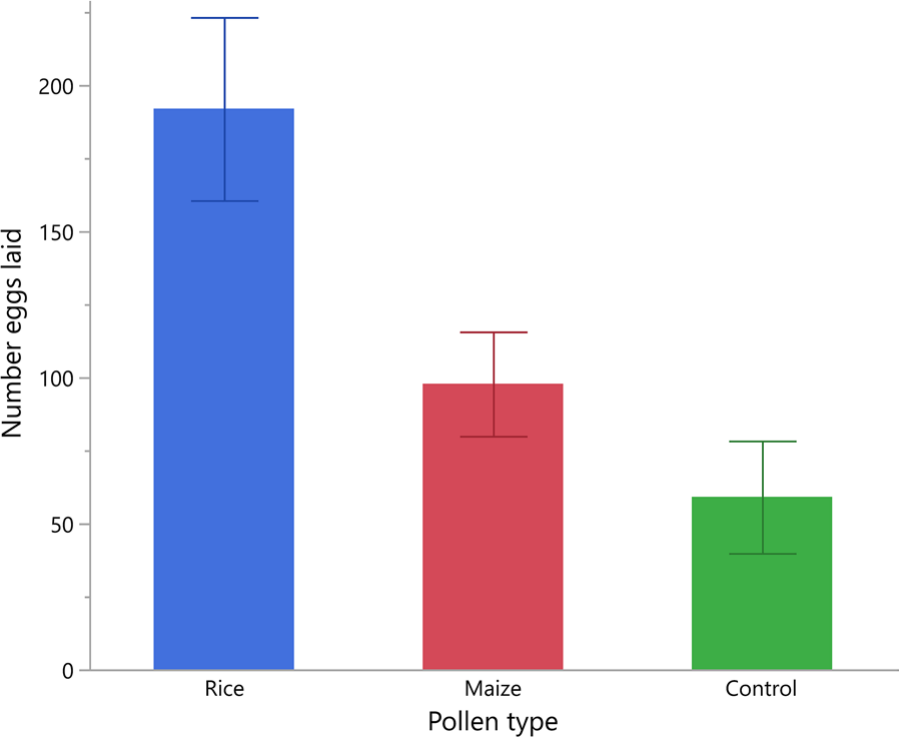
Overall mean number of eggs laid by gravid *Anopheles arabiensis* females across pollen treatments

A generalized linear model (Poisson distribution with over-dispersion) showed that pollen type had a significant effect on the mean number of eggs laid across habitats (L-R chi-square 67.42, *n* = 72, *p* < 0.001) but other variables did not (*p* > 0.05 in all cases). Post-hoc pairwise comparisons showed that significantly fewer eggs were laid on habitats without pollen compared to those with rice or maize pollen (model contrast: *χ*2 > 8.25, *p* < 0.005 in both cases). Also, significantly fewer eggs were laid in habitats with maize pollen than in those with rice pollen (Model contrast: *χ*^2^ = 25.89, *p* < 0.001) (Fig. 4).

### Number of offspring produced and pollen types

Overall, the highest total number of *An. arabiensis* offspring were produced in habitats with rice pollen (*n* = 3422), followed by those with maize pollen (*n* = 2068) and those without pollen (*n* = 1185) (Table 3).

**Table 3:** Mean and total number of *Anopheles arabiensis* offspring produced across pollen types, replicates and compartments

Treatments/replicate	Compartment 1			Compartment 2			Both compartments		
	*N*	Mean offspring,* n* (95% CI)	Total offspring,* n*	*N*	Mean offspring,* n* (95% CI)	Total offspring,* n*	*N*	Mean offspring,* n* (95% CI)	Total offspring,* n*
*Replicate 1*									
No pollen	4	32.3 (– 35.0 to 99.5)	129	4	47 (– 12.1 to 106.1)	188	8	39.6 (8.1–71.1)	317
Maize pollen	4	99.25 (66.3–132.2)	397	4	96.3 (88.4–104.1)	385	8	97.8 (86.0–109.5)	782
Rice pollen	4	152.3 (82.1–222.4)	609	4	181.5 (98.2–264.8)	726	8	166.9 (127.2–206.6)	1335
All treatments	12	94.6 (55.6–133.6)	1135	12	108.3 (65.6–150.9)	1299	24	101.4 (74.7–128.1)	2434
*Replicate 2*									
No pollen	4	60.8 (– 4.5 to 125.9)	243	4	57 (– 4.9 to 119.1)	228	8	58.9 (27.9–89.9)	471
Maize pollen	4	109.3 (73.1–145.4)	437	4	50.3 (– 42.5 to 142.9)	201	8	79.8 (36.5–122.9)	638
Rice pollen	4	75 (– 25.3 to 175.3)	300	4	137.8 (8.9–266.6)	551	8	106.4 (43.6–169.2)	851
All treatments	12	81.7 (52.3–111.0)	980	12	81.7 (37.4–125.9)	980	24	81.7 (57.3–106.1)	1960
*Replicate 3*									
No pollen	4	44.3 (– 37.1 to 125.6)	177	4	55 (– 6.7 to 116.7)	220	8	49.6 (14.2–85.1)	397
Maize pollen	4	69 (– 4.2 to 142.2)	276	4	93 (87.3–98.7)	372	8	81 (53.6–108.4)	648
Rice pollen	4	121.8 (– 38.5 to 282.1)	487	4	187.3 (93.4–281.1)	749	8	154.5 (84.2–224.8)	1236
All treatments	12	78.3 (32.5–124.1)	940	12	111.8 (68.0–155.5)	1341	24	95.0 (65.1–125.0)	2281
*All replicates*	36	84.9 (64.7–105.1)	3055	36	100.6 (77.6–123.5)	3620	72	92.7 (77.6–107.7)	6675

*CI *Confidence interval

**Fig. 5:**
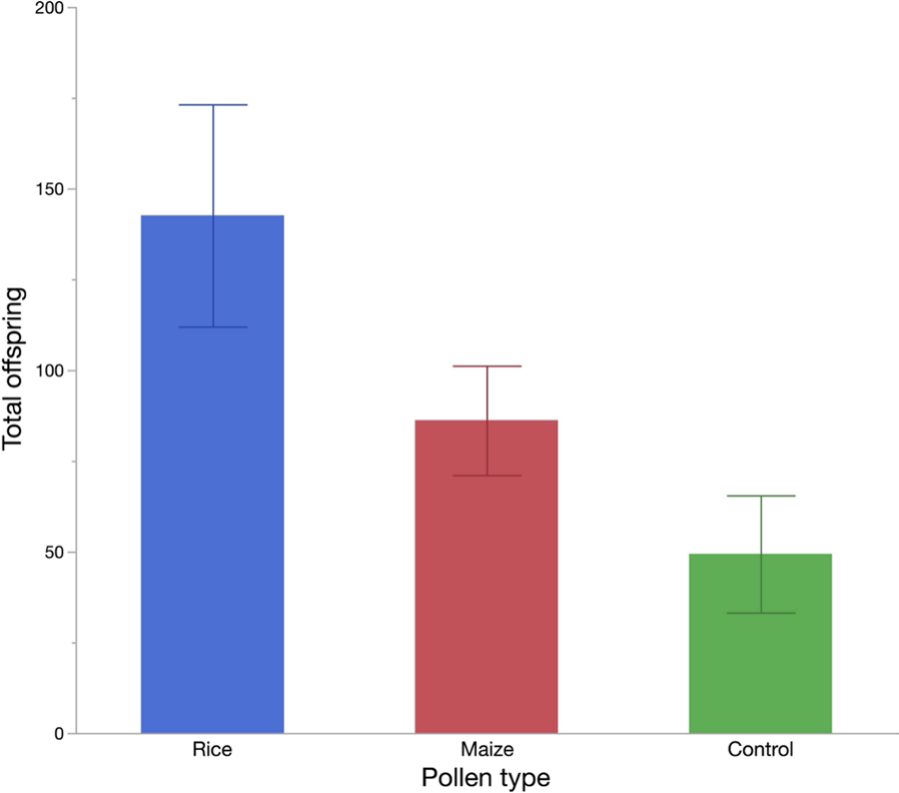
Total number of *Anopheles arabiensis* offspring across pollen type

A generalized linear model (Poisson distribution) showed that pollen types significantly influenced the mean number of offspring produced per habitat (L-R chi-square = 40.93, *n* = 72, *p* < 0.001) but not compartments and replicates. Post-hoc pairwise comparisons showed that significantly fewer offsprings were produced in habitats without pollen than those with rice and maize pollen (Model contrast: *χ*2 > 8.911, *p* < 0.001 in both cases). Additionally, significantly more offspring were produced in habitats with rice pollen than maize pollen (Model contrast: *χ*2 = 12.387, *p* = 0.004) (Fig. 5).

Nominal logistic regression showed that pollen types, replicates, compartment and their interactions had a significant effect on the developmental success of* An. arabiensis* larvae (Table 4). Overall, a higher proportion of larvae successfully developed to the fourth larval instar and pupae in habitats with maize pollen (87.8%), followed by those in control habitats (83.6%) (OR 2.246, *p* < 0.001); the lowest proportion was found in habitats with rice pollen (74.3%) (OR 1.981, *p* < 0.001).

**Table 4:** Effect of pollen type, replicate and compartment on the developmental success of *Anopheles arabiensis* larvae

Source	*df*	L-R Chi-square	*p*-value
Pollen type	2	206.8	< 0.001
Replicate	2	75.2	< 0.001
Compartments	1	11.3	0.001
Pollen type × Replicate	4	80.4	< 0.001
Compartment × Pollen type	2	36.7	< 0.001
Compartment × Replicate	2	8.1	0.0175

**Fig. 6:**
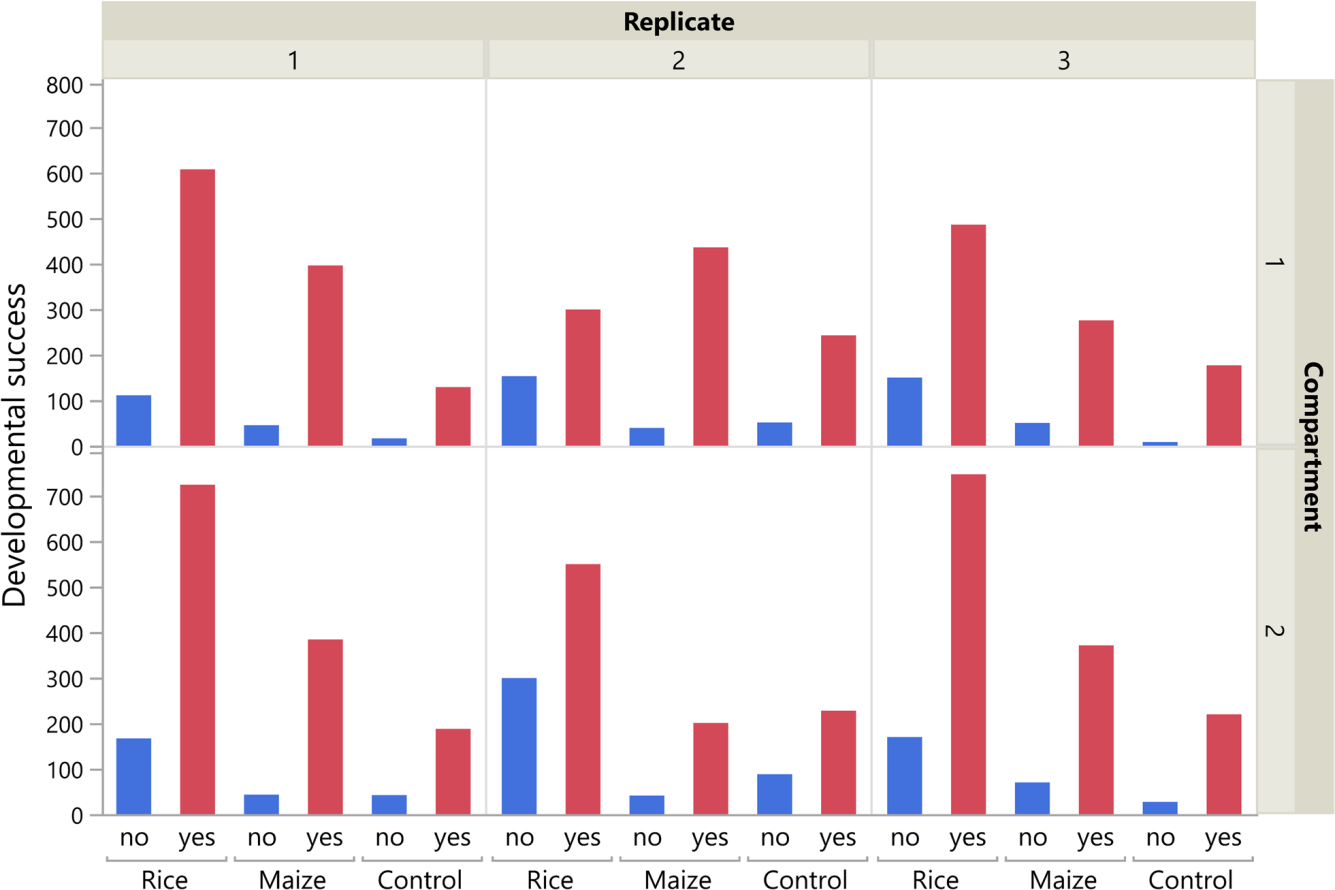
Effects of pollen type, replicate and compartment on the developmental success of *Anopheles arabiensis* larvae

There was no significant difference in larval developmental success in habitats with maize pollen and those without pollen (OR 1.125, *p* = 0.271). Significantly more larvae developed to fourth larval instar and pupae in replicates one and three than in replicate two (Odds ratio > 0.553, *p* < 0.001 in both cases) and in compartment 2 compared to compartment 1 (OR 0.751, *p* < 0.001) (Fig. 6).

**Fig. 7:**
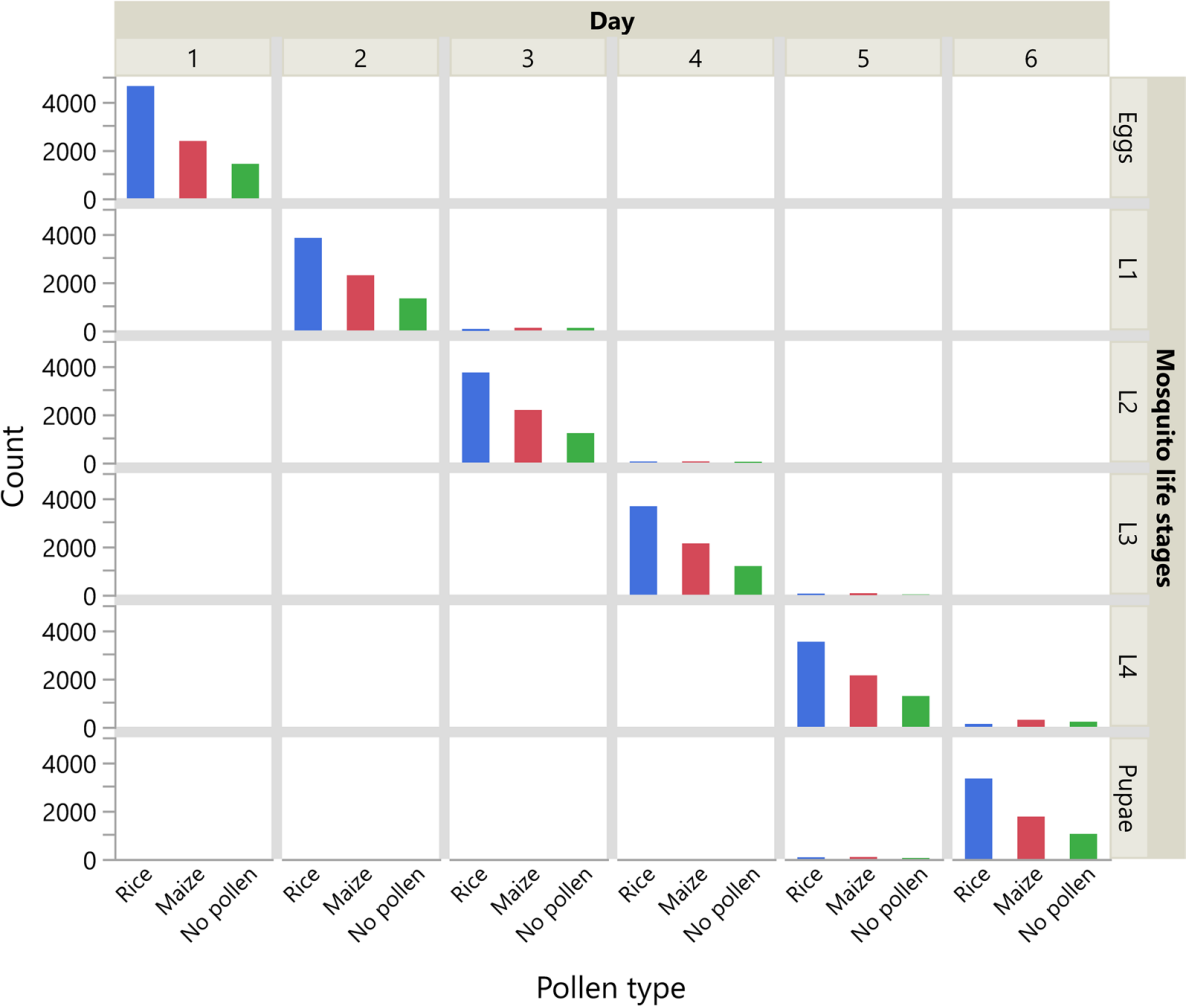
Changes in the number of immature life stages across habitats over the 6 experimental days

Developmental rate significantly varied between pollen treatment groups (L-R Chi-square 297.0, *p* < 0.001), with rice pollen habitats having the lowest proportion of larvae remaining at the fourth instar stage on day 6 (3.50%), compared to maize habitats (85.78%) and pollen-free habitats (83.08%) (Fig. 7). When the daily changes in numbers of the different life stages in the different pollen treatment groups are taken into account, the results reveal that the highest mortality in the rice pollen treatment group occurred at the first instar stage, translating into a very significant interaction term between pollen type and days (repeated measure analysis of variance [ANOVA]: pollen type: *F*_2,57_ = 24.0, *p* < 0.001; day: *F*_1,57_ = 50.4, *p* < 0.001; pollen type × day: *F*_2,57_ = 24.0, *p* < 0.001) (Fig. 7).

## Discussion

This study provides the first insight into how gravid *An. arabiensis* optimize their oviposition strategies in the presence of larval food for their progeny in the form of maize and rice pollen, and the consequence of their choice on the developmental success of their offspring. Using a large semi-field system and through pollen-choice experiments, we demonstrated that pollen type significantly influences the choice of oviposition site of gravid *An. arabiensis*. Females consistently preferred laying their eggs in habitats with rice pollen, followed by habitats with maize pollen and finally by habitats with no pollen.

Previous laboratory-based studies have shown that *An. arabiensis* females are attracted to volatiles emanating from commonly grown African maize *Zea mays* [27] and rice *Oryza* sp. [28]. However, these studies did not compare the response of gravid females in the presence of both maize and rice pollen simultaneously and at a spatial scale relevant to their natural oviposition site search behaviour. The stronger female preference for habitats containing rice pollen over maize pollen observed in this study could be explained by differences in the amounts of attractive volatiles released by the pollen of each plant. Both rice and maize plants and their pollen share (1R)-(+)-α-pinene and nonanal compounds, which have been identified as being attractive to gravid mosquitoes [19, 39]. Thus, it is probable that rice pollen has higher amounts of these compounds than maize pollen. Along the same lines, rice odour may be more appealing to *An. arabiensis* females because it may serve as bifunctional cue simultaneously indicating larval habitats rich in food and, more generally, pointing to water bodies associated with rice paddies, hence potential larval breeding habitats. Finally, because the association between members of the *An. gambiae* complex and rice cultivation is much older than that with maize , which was introduced into Africa in the sixteenth century, females have had a much longer evolutionary time to develop adaptive strategies for detecting rice pollen in breeding habitats. Although these hypotheses remain to be formally tested, our results clearly show that gravid *An. arabiensis* females can distinguish between the pollen of both plants and that they strongly prefer habitats inoculated with rice pollen.

Importantly, under our experimental settings, this strong preference led to a saturation of habitats inoculated with rice pollen, which were all used and had the highest number of eggs laid (average 200.2 eggs). As a result, while more larvae grew in the habitats inoculated with rice pollen than in the others two habitats, developmental success appears to have been hindered by overcrowding, resulting in a lower proportion of larvae reaching the fourth larval instar and pupal stages compared to the proportions in the maize and control habitats. The higher larval mortality observed in crowded habitats may be explained by competition for food and/or space negatively affecting larval growth and survival. This phenomenon has previously been demonstrated in *An. gambiae* s.s. in experiments in which larval density was manipulated [40, 41]. However, in the present study, we did not observe delayed development; rather, habitats with rice pollen had a higher proportion of progeny at the pupal stage than did the other two habitats, suggesting that competition-induced mortality occurred at the early larval stages and that the survivors were the larger and more advanced larvae. The highest developmental success was observed in larvae in the maize pollen habitats, suggesting that larvae in these habitats had enough food for optimal growth, in the form of pollen, and potentially naturally developing protozoan, algae and bacteria. Evidence that such naturally occurring food could sustain a significant number of larvae was highlighted by the successful development of larvae in pollen-free habitats. As per our methodologies, all habitats were prepared 48 h before the start of the experiment with a standard amount of soil (10 g) and well water (20 l) which, combined with natural light, potentially enabled algal growth that in turn generated enough nutrients to sustain the growth of larvae in pollen-free habitats. One limitation of our study was that it was designed primarily to reveal the effects of oviposition site attractions, but not to clearly reveal crowding effects. At present, we cannot dismiss the possibility that differences between rice and maize pollen in terms of digestibility or nutritional value may have contributed to competition being more severe in the rice pollen treatment group. Thus, our experiments would have benefitted from including an additional treatment group in which larvae were fed ad libitum, acting as a positive control for crowding. Alternatively, future experiments could avoid larval overcrowding by using fewer than 40 gravid female mosquitoes per compartment. This would allow a more accurate comparison of larval developmental success between rice pollen- and maize pollen-inoculated habitats.

During the first experiment, differences in the experimental compartments significantly influenced the mean number of eggs laid by female *An. arabiensis*. Gravid females laid a significantly higher number of eggs in compartment 2 than in compartment 1. However, in the second experiment, there was no significant effect of compartment on the number of eggs laid by females, although the developmental success of larvae was higher in the second compartment. While full randomization over treatments was applied within compartments, the experiments were set up sequentially, first in compartment 1 and then in compartment 2, and this could have resulted in a time effect. Random sampling of gravid mosquitoes for each compartment was conducted for one compartment after the other, which may have led to small differences in female quality. The two compartments also differed slightly in their position, which may have affected the angle and amount of light they received. In some analyses, replicate effects were also found, suggesting that the timing of the experiment may have led to differences in environmental conditions, perhaps leading to differences in the physiological state of gravid females and/or larval growth conditions. At present, the exact factor(s) responsible for significant compartment and replicate effects and interactions in some analyses but not others are not fully understood. However, it is noteworthy that their occurrence did not obscure the very strong and consistent pollen treatment effects observed in both experiments.

The semi-field experimental set-up described herein would be ideal for further comparisons of the attractiveness of other pollen types, such as sugar cane [19], or synthetic odour blends optimized to attract gravid *Anopheles* females. Volatile extracts from plants growing on the fringes of aquatic habitats have been shown to be attractive to other species of the *An. gambiae* complex with similar larval ecology, such as *An. coluzzii* from West Africa [42]. This suggests that attraction to rice pollen might well extend to those other important malaria vectors with similar larval ecology.

The fact that gravid *An. arabiensis* females strongly prefer the odour of rice over that of maize should inform novel strategies in which plant volatile compounds are used to lure females to gravid traps [29, 39]. Females could also be lured to lethal larval habitats pre-treated with a larvicide, such as a larval growth regulator, pathogenic fungi, or *Bacillus thuringiensis israelensis* (*Bti*). Such a strategy has been tested for *Culex* mosquitoes, combining the attractant semio-chemical acetoxy hexadecanolide (AHD) and *Bti* larvicide, with promising results [30]. Interestingly, genetically modified (GM) rice strains which express *Bti* have been developed to target rice insect pests, such as the Lepidopteran stem borer [43–45]

The amount of *Bti* found in the pollen of GM rice plants and whether the ingestion of their pollen could negatively affect mosquitoes is currently unknown. It may well be that, in future, the broader use of GM rice and maize crops could reduce the adaptive value of the female oviposition preferences highlighted in this study. In regions of the world where intensive rice cultures are increasingly contributing to high malaria incidence, developing resistant GM rice strains engineered to also express *Bti* toxic to mosquitoes in their pollen could effectively resolve the tense relationship between food security and public health priorities.

## Conclusions

The results of the semi-field experiments reported herein highlight the ability of gravid *An. arabiensis* to distinguish experimental habitats inoculated with rice and maize pollen in their search for a suitable oviposition site. For the first time, the strong preference for rice pollen- and maize pollen-inoculated habitats was expressed at a spatial scale relevant to natural oviposition site choice behaviour. Moreover, the observed patterns of larval developmental success suggest that these preferences are adaptive, even though, in the case of the popular rice field habitats, crowding at the first instar larval stage may have obscured the results. Provided with some minor adjustment of gravid female density, this mesoscale experimental set-up can enable powerful standardized comparisons of natural and synthetic oviposition attractants to the benefit of vector control strategies targeting gravid females or their larvae.

## Data Availability

‘Data supporting the main conclusions of this study are included in the manuscript.

## Abbreviations

AHD: Acetoxy hexadecanolide
Bti: *Bacillus thuringiensis israelensis*
GM: Genetically modified
